# Individual and situational determinants of plastic waste sorting: an experience sampling method study protocol

**DOI:** 10.1186/s40359-021-00596-5

**Published:** 2021-06-03

**Authors:** Valérie J. V. Broers, Melina Van Scharrenburg, Lily Fredrix, Johan Lataster, Ansje J. Löhr, Nele Jacobs

**Affiliations:** 1 Department of Environmental Sciences, Faculty of Science, Open Universiteit, Heerlen, The Netherlands; 2 Department of Lifespan Psychology, Faculty of Psychology, Open Universiteit, Heerlen, The Netherlands; 3 Department of Psychiatry and Psychology, School for Mental Health and Neuroscience, Maastricht University Medical Centre, PO Box 616, 6200 MD Maastricht, The Netherlands

**Keywords:** Plastic waste sorting, Individual, Situational, Pro-environmental behaviour, Determinants, Integrated framework, Experience sampling method, Ecological momentary assessment, Ambulatory assessment

## Abstract

**Background:**

Plastic waste management is one of the most challenging problems of our time. Until now, only 9% of the produced plastics has been recycled. In order to increase recycling, a behavior change towards sorting of plastic waste is needed. Therefore, the main aim of the study is to gain insight in the individual and situational determinants associated with plastic waste sorting behavior. The Integrated Framework for Encouraging Pro-environmental Behaviour will be used as the theoretical framework. This framework assumes that individual egoistic and hedonic values are negatively related to pro-environmental behaviour, whereas individual biospheric and altruistic values are positively related to pro-environmental behaviour. Situational cues can activate these values, resulting in (non) pro-environmental behaviour. Taking the Integrated Framework for Encouraging Pro-environmental Behaviour into account, this study will test the hypothesized associations between individual and situational determinants and plastic waste sorting behavior, using an ecological momentary assessment approach (Experience Sampling Method, ESM).

**Methods:**

A signal-contingent scheme with semi-random intervals will be used for the ESM questionnaire. Over a period of seven consecutive days, an ESM-based smartphone app will prompt participants ten times a day to fill in a short questionnaire containing questions about situational determinants and plastic waste sorting behaviour. Participants will also complete an online questionnaire before and after the study measuring the individual determinants and plastic waste sorting behaviour.

**Discussion:**

ESM has many benefits over traditional surveys, such as improved ecological validity and the possibility to explore temporal relationships. The disadvantages of ESM are mainly related to the burden for the participants and the possibility of reactivity effects. The results will provide insight into the relationship between situational cues, individual values and plastic waste behaviour. The practical implications of the findings of this study can be of interest for policy makers in order to reach plastic waste reduction targets. Furthermore, the situational cues that activate values, which increase or decrease plastic waste sorting, can be targeted in interventions. The results of this study can also be relevant for further research studying and stimulating pro-environmental behaviour in general.

**Supplementary Information:**

The online version contains supplementary material available at 10.1186/s40359-021-00596-5.

## Background

Plastics have revolutionized our daily lives by facilitating the production of products that bring technological advances, energy savings and numerous other societal benefits [1]. This is due to their incredibly versatile properties, such as inexpensiveness, strength and lightweight. For instance, using lighter plastic composites instead of metal in aircrafts results in significant fuel cost savings as well as easier assembly of airplane parts [2]. However, some of the properties of plastics, such as longevity and resistance to degradation, create a major waste management problem [1], especially in developing countries [3]. A global analysis [4] showed that approximately 6300 metric tons of plastic waste have been generated as of 2015, of which only 9% have been recycled. The other 12% were incinerated and 79% have been accumulated in landfills or the natural environment. If the current trends continue, the amount of plastic waste in landfills or the natural environment will be about 12,000 metric tons by 2050 [4]. Many different strategies exist to combat plastic waste pollution. Long-term solutions are aiming at large system changes like moving towards a circular economy and behavioural change [5]. In the current article, we will focus on the behavioural change aspect in the sense that we aim to understand the determinants of pro-environmental behaviour regarding plastics. In our study, pro-environmental behaviour “refers to behaviour that benefits the environment or harms the environment as little as possible” (adjusted from Steg & Vlek[6]). Within pro-environmental behaviour, our focus will be on plastic waste sorting.

### Goals

Many different theories such as the Theory of Planned Behaviour [7], Norm-Activation theory [8] and the Value-Belief-Norm theory [9] aim to explain environmental behaviour, often by focusing on one kind of goal [10]. A goal can be conceptualized as a desired outcome or motive [11]. According to the Goal-Framing Theory of Lindenberg and Steg [10] three types of goals can be distinguished to explain environmental behaviour: hedonic, gain and normative goals. A hedonic goal is aimed at improving the way one feels, for example in terms of seeking direct pleasure or excitement or avoiding effort and negative events [12]. A gain goal is aimed at improving personal resources, for example saving money or increasing status [12]. Finally, a normative goal is aimed at appropriateness, for example behaving the right way or showing exemplary behaviour [12]. Goal-Framing Theory states that multiple goals can be active at the same time and frame how people perceive situations [10]. Usually one of the goals is more salient than the others, however, and is therefore called the goal frame [10]. Goals can be in conflict but it is also possible for goals to strengthen each other [10]. As the goals proposed by Lindenberg and Steg [10] interact, all goals need to be considered to obtain a complete picture of the determinants of plastic waste sorting.

### Values

**Fig. 1:**
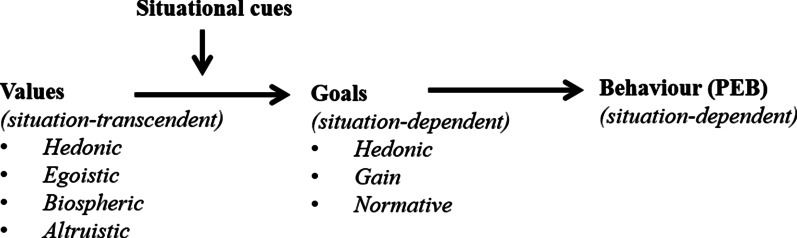
The integrated framework for encouraging pro-environmental behaviour [13]

Changes in goal frames are often not a conscious process, as goals are influenced by other factors such as values that can be activated by situational cues according to the Integrated Framework for Encouraging Pro-environmental Behaviour (Fig. 1) [13]. For instance, the value regarding a concern with nature can be triggered by the presence of a recycling bin, which is a situational cue. As changes in goal frames are often not a conscious process [13], it is difficult for participants to reflect on their goals in a certain situation. Therefore in the current research we will instead focus on the behaviour itself, which results from the goals. Values reflect the overarching goals that people find most important in life in general [14]. They are believed to be relatively stable over time, whereas goals are situation-specific [13]. Hedonic values strengthen the chronic accessibility of hedonic goals, and reflect a self-enhancement concern with improving ones feelings and reducing effort. Egoistic values strengthen the chronic accessibility of gain goals, and reflect a self-enhancement concern with safeguarding or increasing one’s resources. On the other hand, altruistic values strengthen the chronic accessibility of normative goals, and reflect a self-transcendent concern with the welfare of other people. And finally, biospheric values also strengthen the accessibility of normative goals, but they reflect the self-transcendent concern with nature and environment for its own sake. Hedonic and egoistic values are generally more negatively related to pro-environmental behaviour while altruistic and especially biospheric values are generally positively related to pro-environmental behaviour [13]. So for people to engage in pro-environmental behaviour it is important to activate altruistic and biospheric values, and deactivate hedonic and egoistic values with the help of situational cues that influence the relationship between values and goals.

### Situational cues

The Integrated Framework for Encouraging Pro-environmental Behaviour [13] states that specific situational cues can activate values which are more trait-like that in turn influence goals which are more state-like. This is also in line with the Trait Activation Theory (TAT; 15). This theory focuses on the person-situation interaction to explain behavior on the basis of responses to trait-relevant cues found in situations [15]. According to the Integrated Framework for Encouraging Pro-environmental Behaviour [13], the following situational cues affect the likelihood that biospheric values are activated: cues that other people violate or respect norms, behavioural cost and cues that affect the prioritisation of goals [13]. Cues that other people violate norms are expected to weaken normative goals, while cues that other people respect norms would increase the normative goals [13]. An example of these types of cues is clutter or trash in the environment, which indicates that other people have violated the norms. The focus theory of normative conduct [16] predicts that in such situations individuals will act upon the most salient norm.

High behavioural costs, such as the effort, time and costs it takes to engage in the behaviour, are expected to decrease the likelihood that biospheric and altruistic values are activated [13]. According to the low cost hypothesis, people are less likely to act upon normative considerations when the relevant behaviour is associated with relatively high costs [17].

Cues that affect the prioritisation of goals can decrease or increase the likelihood that biospheric values are activated [13]. Situational factors such as time pressure can enhance the need to balance different goals, and result in prioritising hedonic or gain goals (such as time saving), thereby inhibiting the effect of biospheric and altruistic values on behavior [13].

Lindenberg [18] adds to this that the mere presence of other people is one of the most important predictors of normative goal activation. The presence of people in the environment will strengthen one’s own normative goal in comparison to an environment in which no people are present [18]. So we would expect that the presence of other people would increase the likelihood that biospheric and altruistic values are activated. Socially empty environments, such as empty streets, will activate social norms less than socially full environments, and will thus have a higher chance of deviant behavior [19]. So concluding, for people to act pro-environmentally it is important to activate normative goals by triggering biospheric or altruistic values, with the help of situational cues that influence the relationship between values and goals.

So far, we have discussed situational cues that can strengthen or weaken the normative goal directly, but it is also possible for cues to weaken the normative goal indirectly by strengthening hedonic or gain goals [18]. This is possible as according to the Goal-Framing Theory, goals interact and there is usually one goal that is the most pertinent [10]. The most important cues that activate gain goals are cues that indicate that money or competition plays a central role in the environment. Cues such as people wearing a business suit can increase a competitive orientation [18], also called material priming [20].

On the other hand, visceral (intuitive) cues that create affective reactions such as very good or bad smells strengthen hedonic goals, making people automatically more impatient and ready to act on impulse [18]. For example, Li, Moallem, Paller, & Gottfried [21] showed that when people are subliminally primed with an attractive smell, they will also become more impatient in financial transactions. To assess the influence of situational cues that indirectly weaken the normative goals, it is important to take into account gain cues and visceral cues that activate respectively gain and hedonic goals.

When a certain situational cue frequently results in a certain behaviour, the behaviour can become a habit [22]. Habits are automatic responses to specific cues, in order to obtain certain goals [22]. Because of their automatic nature, they do not need mental effort and are therefore very efficient [23]. To increase the efficiency of interventions, it is thus important to assess which situational cues that influence plastic waste sorting, are associated with habit strength. It is especially relevant to elucidate the situational cues that predict pro-environmental behaviour in individuals with a strong habit of recycling plastic, as it would be most efficient to target these cues in order to change behaviour. Habit strength of plastic waste sorting will therefore be tested exploratory as a moderator for the situational cues predicting plastic waste sorting.

### Experience sampling method

In the current study, we measure the situational cues that activate values regarding plastic waste sorting in the natural habitat of people, using the Experience Sampling Method (ESM). Experience sampling, also called Ecological Momentary Assessment or Ambulatory assessment, is a type of diary method where information is collected about the context and content of the daily life of individuals [24]. ESM studies are conducted in close temporal proximity to behaviour, and therefore reduce the disadvantages of self-reports, such as memory and estimation problems [25]. ESM studies are known for their improved ecological validity, as ESM ensures an assessment in the natural setting [25]. A third advantage of ESM is that temporal relationships among variables can be explored as independent variables can be measured at an earlier time point compared to dependent variables [25]. In the current study, we are therefore able to predict plastic waste sorting by using the situational cues that were measured at the time-point that precedes the behaviour.

As people self-monitor their behaviour in ESM studies, however, it is possible that the monitored behaviour changes due to a reactivity effect (25; 26). Measurement reactivity entails systematic biases in methods and procedures that affect the validity of the data. The extent of the reactivity depends on certain aspects such as the awareness of the behaviour, explicit feedback and the perceived desirability of the behaviour [25]. Social desirability can be problematic, as it can arise when participants attribute socially desirable values to themselves and reject socially undesirable values [27]. In this study participants might over-report altruistic and biospheric values and plastic waste sorting as they are perceived as socially desirable. To our knowledge, the current study is the first ESM study concerning plastic waste sorting with the aim of understanding the situational cues that determine normative goal activation, taking into account the social desirability and the reactivity effect.

### Hypotheses

Based on the findings in the literature [13, 18], we expect hedonic and egoistic values to be negatively associated, while altruistic and biospheric values are positively associated, with plastic waste sorting measured with the ESM questionnaire. We also expect that situational cues will moderate this relationship. For biospheric and altruistic values we expect the following situational cues to weaken the relationship between altruistic/biospheric values and plastic waste sorting: behavioural cost (further distance to recycling bin, no presence of recycling bin), situational factors that affect the prioritisation of goals (time pressure, ego-depletion), environmental cues that violate norms (clutter, trash) and the absence of people. Furthermore, we expect situational gain cues (business suits, competitiveness) to enhance the relationship between egoistic values and plastic waste sorting. Finally, we expect situational visceral cues (i.e. very high and very low vs. neutral scores on attractiveness surroundings, smell, affect) to enhance the relationship between hedonic values and plastic waste sorting. Habit strength of plastic waste sorting will be consequently tested exploratory as a moderator for the situational cues that predict plastic waste sorting measured with the ESM questionnaire. And finally, we expect plastic waste sorting measured with the survey to increase after the data-collection in comparison to prior to the data-collection as an effect of the monitoring of this behaviour.

## Methods

### Design

The study is observational with a mixed design (within-participants: ESM measures and between-participants: survey measures).

### Participants

Participants eligible for this study are Dutch-speaking people of 18 years or older living in the Netherlands and possessing a smartphone. Participants completing at least 1/3 of the questionnaires they receive with signals on their smartphone (also called beeps) will be included in the final analysis, in line with the guidelines proposed by Delespaul [28]. In order to test the feasibility, a pilot study was conducted including 23 participants. Nine participants did not fill out the baseline questionnaire, resulting in 14 participants. Of these 14 participants, 7 participants filled out at least 1/3 of the total beeps ranging from 44 to 92% of the maximum beeps. The outcome beeps that can be used for the analyses (plastic waste sorting/non-plastic waste sorting) of these people ranged from 2 to 50, with plastic waste sorting being reported 0 to 11 times per person. Two smaller pilots (N = 10 and N = 6) were conducted previously to test the feasibility and comprehensibility of the study. Minimum sample size for establishing medium-sized effects (Beta = 0,5) was estimated at n = 250 (logistic multiple regression, power ≥ 0.80 for all random and fixed effects, a skewed level 1 predictor, a standard normal level 2 predictor, an interaction, 10 simulations) with a shiny R web application and its R packages lme4, simglm and paramtest [29]. Intercept and slope variance for the random effects were based on the pilot data.

### Data collection and procedure

For the main study, participants will be recruited through social media, networks, flyers in supermarkets, universities, sports clubs etc. Students of the Open University can also participate in exchange for study credits. Participants can sign up by emailing the researchers. On day 1 of the research period, participants will receive an email that includes the information letter, the guide to install the ESM-based smartphone application and the link to the baseline online survey. From day 2 until day 8, participants will receive 10 smartphone beeps per day that include the ESM questions. And finally, on day 9 participants will receive a prompt on the application with a link to the second and final online survey. See Table 1 for an overview of the procedure for the participants.

**Table 1:** Procedure of the study for participants per day

Day 1
Fill out online survey 1 (15 min)
Install application and look at demo-questions
Day 2 until 8
Fill out questions application after signal on smartphone (2 min × 10 per day)
Day 9
Fill out online survey 2 (2 min)

All participants will use their own Android or iPhone smartphone. The software used is ‘Real Life Exp’ Version 2.8.26 [30]. Participants will be prompted via standard push notifications. It is not necessary to have an internet connection at all times, beeps will also be received offline, and data are transferred once a connection is made. Participants will be asked to have both the sound and vibration on for their notifications, but they are able to turn notifications off.

This study was approved by the Research Ethics Committee (cETO) of the Open University (decision #U2019/03119/MQF). Participants will be informed about the aim of the study with an information letter, and written informed consent will be obtained online at the start of the survey. Data will be stored on a secured drive that is only accessible by the main researcher. Data will be stored for 10 years on the secured drive. Each participant will create a personal code combining the first two letters of their first name with the year that they were born, to ensure anonymity. The data collected by the surveys and the ESM application will be combined with the help of this anonymous code. The study is preregistered at the Open Science Framework (https://osf.io/numsd) on the 9th of March 2020. If amendments will be made to the protocol, they will be registered at the Open Science Framework. Furthermore, this research proposal was peer reviewed by a multidisciplinary internal OUNL review committee consisting of senior researchers and has been awarded a grant from the OUNL research program DALI (Digital Accessible Learning Innovation), theme ‘Safety in urban environments’.

### Survey

The online survey on day 1 and day 9 will be conducted via LimeSurvey, all items of this survey can be found in an additional file (see Additional file 1: Appendix 1). The first survey will be composed of eight sections, the second survey will be composed of only one section (pro-environmental behaviour) of the former eight. The following sections will be used in the analysis:

- *Demographic characteristics* included gender, age, education, civil status, household composition, income and neighbourhood.
- *Habit strength* of separating plastic waste will be assessed with one 12-item questionnaire (Self-Report Habit Index; 23). The SRHI is currently the most commonly used measure of habit strength in health behaviours [31]. The scale also possesses a high internal reliability, as Cronbach alphas are often greater than 0.90 (e.g. 31, 32). An example item is: “Sorting plastic waste is something that I do without thinking.” The items will be measured on a 5-point Likert scale, ranging from 1 (strongly disagree) to 5 (strongly agree). The scores will be recoded such that high values indicated strong habits. A mean of the 12 habit items will be calculated.
- *Value orientations* will be assessed with a 16 item-questionnaire by means of a short version of Schwartz’s value scale [14] developed by De Groot and Steg [33] and adapted by Steg, Perlaviciute, van der Werff and Lurvink [34]. The scale has extensively been tested and validated in various studies (i.a. 34, 35). The questionnaire comprises four value clusters: biospheric (4 items), altruistic (4 items), egoistic (5 items) and hedonic (3 items). Participants will be asked to rate the importance of these values as guiding principles in their lives, and will be urged to vary the scores to ensure they only rated few values as extremely important. An example item for biospheric value orientation is: “respecting the earth (living in harmony with other species)”. An example item for altruistic value orientation is: “social justice (correcting injustice, care for the vulnerable and weak)”. An example item for egoistic value orientation is: “social power (control over others, dominance)”. An example item for hedonic value orientation is: “enjoying life (enjoying food, sex, leisure, etc.)”. The items will be measured on a 9-point scale ranging from − 1 (opposed to my principles), 0 (not important) to 7 (extremely important). A mean will be calculated for each value cluster.
- *Social desirability* will be assessed with a 10-item questionnaire (shorted version of the Marlowe-Crowne Scale M-C 2 [10], 36] translated to Dutch [37], which has been well validated [38]. An example item is: “I never hesitate to go out of my way to help someone in trouble.” The items will be measured on a True or False scale. For each statement a person can score 1 point if he indicates social desirability. The total scale score will be calculated by adding the points that people obtain, so it ranges from zero to ten.
- *Pro-environmental plastic waste behaviour* will be assessed with a 7-item questionnaire developed for the current study and measuring similar items as the ESM measure regarding plastic pro-environmental behaviour. The questionnaire consists of the following items: “How often have you used plastic packaging in the past week?”, “In case you used a plastic packaging, how often did you throw it in a separate waste bin in the past week?”, “In case you used a plastic packaging, how often have you thrown it in a regular waste bin in the past week?”, “In case you used a plastic packaging, how often did you throw it on the ground in the past week?”, “In case you used a plastic packaging, how many times have you left it somewhere in the past week?”, “In case you used a plastic packaging, how often have you reused it in the past week?”, “In case you used a plastic packaging, how often have you kept a plastic package for later disposal in the past week?” The items will be measured on a 7-point Likert scale, ranging from 1 (not at all) to 7 (to a very high degree).

### ESM questionnaire

A signal-contingent scheme with semi-random intervals will be used for the ESM questionnaire. The ESM period will last for 7 days and participants will receive 10 beeps per day in bursts between 7.00 AM and 9.30 PM. The first beep is randomly scheduled between 8.00 AM and 4.30 PM. The 9 consecutive beeps are each randomly scheduled between 30 and 45 min from their preceding beep. A time window is scheduled between beeps, so that there are at least 15 min between consecutive beeps. The six sampling schedules will be varied over the different days of the week (Monday, Tuesday, Wednesday, Thursday, Friday, Saturday, Sunday) to ensure that the total dataset contains observations from several timeslots for each day of the week. Following the initial notification, the ESM questionnaire will be available to the participant for 15 min.

Following the pilot, we estimate the completion time for each questionnaire to range from 2 to 4 min. Questionnaire length does not vary as a function of certain item responses as filler questions have been used to keep burden equal independent of item branching. All items of the ESM questionnaire can be found in an additional file (see Additional file 2: Appendix 2). The ESM questionnaire consists of 28 multiple choice items, including the following items that will be used in the analysis:

- *Momentary affect* (situational cue) will be assessed with 4 positive and 4 negative affect items derived from the Positive And Negative Affect Schedule (PANAS, 39; 40). The PANAS is considered to be a reliable and valid measure [41]. Items were selected to assess a broad range of affect across the dimensions of ‘valence’ (positive–negative) and ‘arousal’ (high–low) [42]. Example items are: “I feel cheerful” (positive affect, high arousal), and “I feel down” (negative affect, low arousal). The items will be measured on a 7-point Likert scale, ranging from 1 (not at all) to 7 (very much so) and will be presented in a random order. A mean will be calculated for positive affect and negative affect.
- *Competitiveness* (situational cue) will be assessed with a single item: “I feel competitive”. The item will be measured on a 7-point Likert scale, ranging from 1 (not at all) to 7 (very much so).
- *Time pressure* (situational cue) will be assessed with a single item “I am in a hurry”. The item will be measured on a 7-point Likert scale, ranging from 1 (not at all) to 7 (very much so).
- *Ego-depletion* (situational cue) will be assessed with a single item “I have a lot of mental energy” derived and adapted from the State Self-Control Capacity Scale [43]. The item will be measured on a 7-point Likert scale, ranging from 1 (not at all) to 7 (a lot) and rescored in order for high values to reflect a high score on ego-depletion.
- *Presence of other people* (situational cue) will be assessed with a single item: “With whom am I?”. Answers were categorized into 9 different options, examples being “nobody”, “partner”, “children”, etc. Presence of other people will be recoded in 0 for nobody and 1 for all the other categories.
- *Gain cue* (situational cue) will be assessed with a single item: “Are there people present wearing business clothes?”. The item will be measured on a 7-point Likert scale, ranging from 1 (none) to 7 (a lot).
- *Attractiveness surroundings* (situational cue) will be assessed with a single item: “How beautiful is it here?”. The item will be measured on a 7-point Likert scale, ranging from 1 (not at all beautiful) to 7 (very beautiful). The item will be recoded in 1 = 4; 2 = 3; 3 = 2; 4 = 1; 5 = 2; 6 = 3; 7 = 4, as we are interested in extreme vs neutral scores.
- *Presence of green nature* (situational cue) will be assessed with a single item: “How much green do I see? You can think of plants, flowers, trees, grass etc.”. The item will be measured on a 7-point Likert scale, ranging from 1 (none) to 7 (a lot).
- *Smell surroundings* (situational cue) will be assessed with a single item: “How pleasant does it smell here?”. The item will be measured on a 7-point Likert scale, ranging from 1 (not pleasant at all) to 7 (very pleasant). The item will be recoded in 1 = 4; 2 = 3; 3 = 2; 4 = 1; 5 = 2; 6 = 3; 7 = 4, as we are interested in extreme vs neutral scores.
- *Presence of clutter* (situational cue) will be assessed with a single item: “How much clutter is present?”. The item will be measured on a 7-point Likert scale, ranging from 1 (none) to 7 (a lot).
- *Presence of waste* (situational cue) will be assessed with a single item: “How much waste is present?”. The item will be measured on a 7-point Likert scale, ranging from 1 (none) to 7 (a lot).
- *Presence of plastic recycling bin* (situational cue) will be assessed with a single item: “Can I separate plastic waste nearby?” Answers will be categorized into 3 different options, “yes”, “no” and “I don’t know”. Answer options “no” and “I don’t know” will be recoded to 0, “yes” will be recoded to 1.
- *Distance to a plastic recycling bin* (situational cue) will be assessed with a single item: “How far away is the plastic recycling bin?” The item will be measured on a 7-point Likert scale, ranging from 1 (not far away at all) to 7 (very far away). This item will only be presented to participants who indicated that there is a plastic recycling bin nearby, other participants will receive a filler question (“Which other type of waste can I throw away separately here?”) to keep the burden equal for all participants.
- *Plastic usage* will be assessed with a single item: “Since the last beep, have I used something packaged in plastic? (if this is the first beep of the day, think of the period since you got up)”. Answers will be categorized into 2 different options, “yes” and “no”.
- *Plastic waste sorting* will be assessed with a single item: “What have I done with the plastic packaging? (multiple answers possible in case of multiple plastic packages)”. Answers will be categorized into 7 different options: “I threw it away in a separate plastic bin”, “I threw it away in a regular bin”, “I threw it on the ground”, “I left it behind”, “I am still using it”, “I kept it to use again at a later time” and “I saved it to throw away at a later time”. This item will only be presented to participants who indicated that they used something in plastic packaging since the last beep, other participants will receive a filler question (“Since the last beep, have I used something packaged in glass?”) to ensure equal burden for all participants. This item will be recoded in 1 for “I threw it away in a separate plastic bin" and 0 for “I threw it away in a regular bin”, “I threw it on the ground”, “I left it behind”. The other options will be recoded as missing values, since we want to compare non-environmental behaviour to plastic waste sorting in this study, and some of the other options include other types of pro-environmental behaviour like re-using.

### Data-analysis

We will use a multilevel logistic model to analyze the data. The data has a two level structure: repeated measurements (level 1) nested within individuals (level 2). The dependent, binary variable is sorting plastic waste [1] or not sorting plastic waste (0; throwing in a normal waste bin, throwing on the ground or leaving the plastic somewhere) measured with ESM. Predictors will be the values (hedonic, egoistic, biospheric, altruistic). The situational factors will be tested as moderators between the specific values and plastic waste sorting. A correction will be applied to take the number of performed tests in account.

We will also use a multilevel logistic model to analyze the results for habits. The data has a two level structure: repeated measurements (level 1) nested within individuals (level 2). The main dependent, binary variable is sorting plastic waste [1] or not sorting plastic waste (0; throwing in a normal waste bin, throwing on the ground or leaving the plastic somewhere) measured with ESM. All situational factors will be predictors, and habit strength will be tested as a moderator for the situational factors that significantly predict plastic waste sorting. A correction will be applied to take the number of performed tests in account.

A repeated measures ANOVA analysis will be performed to see if plastic waste sorting measured by the online survey increases from before the study (T0) to after the study (T1).

## Discussion

The aim of this study is to gain insight in plastic waste sorting behaviour, and its individual and situational determinants, using the Experience Sampling Method. The innovative aspect of this study lies in the naturalistic setting of the study using the Experience Sampling Method and the inclusion of both individual and situational determinants to predict plastic waste sorting behaviour. The Experience Sampling method, consisting of multiple measurements in the natural environment, is a definite strength of the study, as previous studies used one-time correlational measures when assessing determinants of pro-environmental behaviour [44]. ESM has many benefits over traditional surveys, such as improved ecological validity and the possibility to explore temporal relationships [25]. This possibility allows us therefore to measure the situational cues at the preceding time-point of plastic waste sorting, while ensuring a high ecological validity of the data.

The measures in this study consist of self-reports. Self-reports are based on the assumption that people know their thoughts, feelings and behaviours and can report on them accurately [44]. Disadvantages of self-reports are related to misunderstandings of questions, inability to access the information that is required, the limits of memory and social desirability [25]. Many self-report issues can be resolved however by asking questions in close temporal proximity to the event of interest [25]. It minimizes the multiple meanings of questions, reduces memory and estimation problems and facilitates access to episodic detail. As ESM studies are conducted in close temporal proximity to behaviour, the disadvantages of self-reports are considerably reduced. Social desirability will also be taken into account as this measure has been included in the study.

However, compared to traditional surveys, the burden on participants is considerably higher because of the repeated questions [45]. To address this issue, the questionnaire that was designed for this study is short and takes only a couple of minutes to fill out. Nevertheless, recruitment of participants can be difficult as ESM studies are time-consuming [45]. Therefore, we will recruit participants through multiple platforms, such as social media, networks, flyers in supermarkets, universities and sports clubs. Students of the Open University can participate in exchange for study credits. Other participants will be incentivized by providing the results of the study and underlining the importance of their participation to help creating a more sustainable planet. The pilot study showed that the commitment of participants may not be strong enough, as a considerable amount of participants were lost due to missing baseline data or too much missing beep data. Maintaining ongoing personal contact will therefore be important as this retains participants more than monetary incentives or dependence upon goodwill towards science [45]. To encourage participants to fill in the beeps, a motivational message in the final beep of each day was included, encouraging them to continue. To avoid missing baseline data, additional prompts were added in the ESM application reminding participants to fill out the baseline survey. These adaptations should increase participant compliance and retention.

The findings of this research can be theoretically relevant as, for the first time, the Integrated Framework for Encouraging Pro-environmental Behaviour [13] will be tested in the context of plastic waste sorting. The results will provide insight into the relationship between situational cues, individual values and plastic waste sorting behaviour. The practical implications of the findings of this study can be of interest for policy makers in order to reach waste reduction targets. The situational cues that activate values which increase or decrease plastic waste sorting can be targeted in interventions for instance. A behaviour change technique that utilizes situational cues is nudging. Nudging refers to interventions that organize the choice architecture in order to change people’s behaviour in an automatic way without forbidding any options or significantly changing their economic incentives [46]. If we can distinguish which situational cues link to plastic waste sorting behaviour for people with strong habits, future interventions could specifically target these cues as they will be most likely to turn plastic waste sorting into a habit. So both theoretically as well as practically, this study can have important implications regarding the understanding and altering of plastic waste sorting behaviour and pro-environmental behaviour in general.

## Supplementary Information

**Additional file 1.** Appendix 1 - Survey items.

**Additional file 2.** Appendix 2 - ESM items.

## Data Availability

Not applicable. Upon completion of this study, the datasets generated and analysed will be available in the Open Science Framework repository, https://osf.io/numsd.

## Abbreviation

ESM: Experience sampling method
