# Supplementary material for: Individual and situational determinants of plastic waste sorting: an experience sampling method study protocol
Source: BMC Psychol. 2021 Jun 3;9:92. doi: 10.1186/s40359-021-00596-5 (PMC8173730; doi:10.1186/s40359-021-00596-5)
Supplement: Supplementary file 1 — Additional file 1. Appendix 1 - Survey items. [file 40359_2021_596_MOESM1_ESM.pdf]

ESM-questions

Welcome to this study. From tomorrow onwards you will be asked to fill out a short survey 10 times per day for 7 days. At these moments the app will give you a signal on your phone. Fill out the questions as soon as possible after this notification. Many thanks in advance and good luck with this research.

	Not at all				Very much so		
**Momentary affect**							
I feel cheerful	1	2	3	4	5	6	7
I feel enthusiastic	1	2	3	4	5	6	7
I feel content	1	2	3	4	5	6	7
I feel happy	1	2	3	4	5	6	7
I feel anxious	1	2	3	4	5	6	7
I feel irritated	1	2	3	4	5	6	7
I feel guilty	1	2	3	4	5	6	7
I feel down	1	2	3	4	5	6	7
**Competitiveness**							
I feel competitive	1	2	3	4	5	6	7
**Time-pressure**							
I am in a hurry	1	2	3	4	5	6	7
**Ego-depletion**							
I have a lot of mental energy	1	2	3	4	5	6	7

Location

Where am I?

- at home
- at someone else’s house
- at work/school/education
- in nature
- in the supermarket
- at the gym / sports club
- on public transportation

- in a restaurant / café / pub
- in the cinema/theater/musum
- in the street
- somewhere else

Presence of other people
With whom am I? More than one answer is possible.

- Nobody
- Partner
- Child(ren)
- Parent(s)
- Brother(s) / sister(s)
- Other family / family-in-law
- Co-worker(s) / co-student(s)
- Friend(s) / acquaintance(s)
- Stranger(s) / other(s)

Gain cue
Are there people present wearing business clothes?

None					A lot	
1	2	3	4	5	6	7

Fealing at ease

	Not at all					Very much so	
I feel at ease	1	2	3	4	5	6	7
I can be myself	1	2	3	4	5	6	7

Attractiveness surroundings
How beautiful is it here?

Not at all beautiful					Very beautiful	
1	2	3	4	5	6	7

Presence of green nature
How much green do I see? You can think of plants, flowers, trees, grass etc.

None						A lot
1	2	3	4	5	6	7

Smell surroundings
How pleasant does it smell here?

Not pleasant at all						Very pleasant
1	2	3	4	5	6	7

Presence of clutter
How much clutter is present?

None						A lot
1	2	3	4	5	6	7

Presence of waste
How much waste is present?

None						A lot
1	2	3	4	5	6	7

Type of waste
Is this waste …. (more than one answer is possible)

- Not applicable (there is no waste)
- Organic (food, garden, other green waste)
- Plastic
- Paper / cardboard
- Glass
- Other

Presence of plastic recycling bin
Can I separate plastic waste nearby ?

- Yes
- No
- I don’t know

When the answer is “no” or Ï don’t know” for presence of plastic recycling bin:
Distractor
Which other type of waste can I throw away separately here?

- None
- Organic (food, garden, green waste)
- Paper / cardboard
- Glass
- Other

- I don’t know

When the answer is “yes” for presence of plastic recycling bin:
Distance to a plastic recycling bin

	Not far away at all					Very far away	
How far away is the plastic recycling bin?	1	2	3	4	5	6	7

Activity
What was I doing (before the signal)? More than one answer possible.

- Eating / drinking / snacking
- Nothing / resting / reading / watching television
- Working / studying
- Sports
- Travelling / on the way / on the go
- Shopping / groceries
- Self-care / caring for other(s)
- Going out (e.g. cinema, theatre)
- Hobby
- Cooking
- Walking / hiking
- Household chores
- Other

Plastic usage
Since the last beep, have I used something packaged in plastic? (if this is the first beep of the day, think of the period since you got up)
- Yes
- No

When the answer is “yes” for plastic usage:
Plastic waste sorting
What have I done with the plastic packaging? (multiple answers possible in case of multiple plastic packages)

- I threw it away in a separate plastic bin
- I threw it away in a regular bin
- I threw it on the ground
- I left it behind

- I am still using it
- I kept it to use again at a later time
- I saved it to throw away at a later time

When the answer is "no" for plastic usage:
Glass usage
Since the last beep, have I used something packaged in glass? ? (if this is the first beep of the day, think of the period since you got up)

- Yes
- No

**Disturbance**	Not at all						Very much so
This beep bothered me	1	2	3	4	5	6	7
